# Health Literacy in the Canton of Zurich: First Results of a Representative Study

**DOI:** 10.3390/ijerph182312755

**Published:** 2021-12-03

**Authors:** Elena Guggiari, Rebecca Jaks, Fabian Marc Pascal Berger, Dunja Nicca, Saskia Maria De Gani

**Affiliations:** 1 Careum Foundation, Health Literacy Department, Pestalozzistrasse 3, 8032 Zurich, Switzerland; Elena.Guggiari@careum-hochschule.ch (E.G.); rebecca.jaks@careum.ch (R.J.); Fabian.Berger@careum-hochschule.ch (F.M.P.B.); 2 Research Department, Careum School of Health, Gloriastrasse 18a, 8006 Zurich, Switzerland; 3 Epidemiology, Biostatistics and Prevention Institute, University of Zurich, 8001 Zurich, Switzerland; dunja.nicca@uzh.ch

**Keywords:** health literacy, health information, health determinants, HLS-EU-Q, federalist health system

## Abstract

Managing health information and services is difficult for nearly half of the population in Switzerland. Low health literacy has been shown to result in poorer health and health outcomes as well as a higher utilization of health services. To date, studies on health literacy in Switzerland have focused on a national level. However, Switzerland is a federal state with 26 cantons and a strongly decentralized health system. Therefore, the aim of this study is to understand how health literacy is distributed within the population of the canton of Zurich specifically, and to develop methods to determine whether an individual has a higher or lower level of health literacy. There were a total of 1000 participants in this representative study. Data was collected by an adapted version of the HLS-EU-Q47 and additional sociodemographic questions. The majority (56%) of the reported difficulties concerned accessing, understanding, appraising, and applying health information. The findings confirm that health literacy follows a social gradient, whereby financially deprived individuals and those with a low educational level report lower health literacy. The need for action to strengthen the health literacy of these population groups is therefore urgent. Interventions should pay particular attention to these vulnerable groups and tailor resolutions to their needs and preferences.

## 1. Introduction

Health literacy is defined as people’s knowledge, motivation and competence to access, understand, appraise, and apply health information in order to make decisions in everyday life concerning healthcare, disease prevention and health promotion to maintain or improve quality of life [1,2]. In other words, health literacy allows a person to navigate in a continuum, ranging from being a patient in the healthcare setting, to a person at risk of a disease, and to a person focused on health-promotion efforts. The population is increasingly required to play an active role and take responsibility for its own health and that of other people. At the same time, more self- and co-determination is demanded from people. To take on the responsibility develop the ability to perform the associated tasks, all people are dependent on certain abilities and skills, and hence require sufficient health literacy.

Research on health literacy has been expanding in the last years. A recent representative study conducted in Switzerland [3] shows that almost half of the Swiss population (49%) reports frequent difficulties in dealing with health information and that health literacy has decreased slightly from 2015 to 2020 [3]. In addition, the results consistently show that the greatest challenges lie in the area of disease prevention, compared to the areas of health promotion and healthcare. Furthermore, low health literacy is closely linked to financial deprivation and a lack of social support, whilst education level, employment status and migration background also have a small influence on health literacy. A study carried out in Europa found similar results, suggesting the existence of a social gradient for health literacy [4]. In particular, a strong correlation with financial deprivation has been identified, indicating lower health literacy with increasing financial deprivation, strong and positive correlations with social status and education, and correlations with age and gender. A recent study in Germany [5] also confirmed that health literacy is not equally distributed among the population, i.e., people with lower education, lower social status, a migration background, elderly people and chronically ill tend to report lower health literacy. Similar to findings in other European countries [6], the study conducted in Switzerland also shows that low health literacy can have negative consequences on health behavior and health status as well as on the use of the health system. In general, health-promoting behavior is positively associated with higher health literacy. In contrast, people that experience more difficulties in managing health information and services often feel less healthy and tend to use the health system more often [3].

In Switzerland and many other countries, health literacy is often investigated on a national level. However, the “Health Literacy Survey Switzerland 2015” (HLS_15_-CH) has already revealed some regional differences concerning the distribution of health literacy [7]. Considering that Switzerland has a strongly decentralized federal system based on 26 different cantons, each of them having different health systems and health policies, regional, and more specifically, cantonal differences deserve specific attention. Therefore, the objective of this study was to acquire representative data on health literacy of the population of the canton of Zurich. Specifically, we aimed to understand the way health literacy is distributed within the population of Zurich and how to identify whether an individual has a higher or lower level of health literacy. For this purpose, data on health literacy of the population of the canton of Zurich was collected within the “Health Literacy Survey Zurich” (HLS-ZH-18).

## 2. Materials and Methods

### 2.1. Study Population

A representative survey among the population of the canton of Zurich was conducted by the Careum Foundation and the Department of Health of the canton of Zurich. Some of the data have been analyzed and published in another context [8,9]. The sample included a total of 1000 respondents (age ≥ 18 years). The present sample size was sufficient to conduct population and subgroup analyses. The sampling error was 3.2. The sample was drawn through the random selection of 100 cantonal sampling points where quotas were set on site, considering gender and age. Ten interviews were conducted per sampling point. The basis for the drawing of the sampling points were communities with at least 1000 residents. Larger municipalities had several sampling points (one per 1000 persons).

Data were collected by means of computer-assisted personal interviews (CAPI) between November and December 2018 (mean duration was 30.3 (+6) minutes) by a third party (gfs.bern AG, research institute, Bern, Switzerland). Trained interviewers randomly interviewed pedestrians in the German language, whereby interviewers were free to choose where they contacted the participants. Participants provided their informed consent to participate in the study after having been verbally informed about the objectives, framework, conditions, and data protection measures. The whole process was in line with the legal and association requirements for data protection and personal rights (VSMS) and therefore an ethical approval was deemed as not necessary.

### 2.2. Questionnaire

Health literacy was measured using the questionnaire of the national survey HLS_15_-CH [6], which in turn is based on the original European questionnaire, the HLS-EU-Q47 [10]. The 47 items provided were adapted for Switzerland and have been used also for this study, permitting comparisons between the Swiss population and the population of the canton of Zurich. Minor changes have been made in this study for questions concerning sociodemographic characteristics and health behavior. However, the core questionnaire remained unchanged to ensure the greatest possible comparability between the two studies. In agreement with the Swiss Federal Office of Public Health, the original questionnaire from the HLS-EU that had been adapted for the Swiss national study (HLS_15_-CH) had not been separately validated for Switzerland. Nevertheless, as the instrument was based on the HLS-EU-Q47, which was pre-tested for validity in three focus groups (in Greece, Ireland, and the Netherlands) and field-tested with 50 computer-assisted face-to-face interviews in two countries (*n* = 99 in Ireland and the Netherlands), it was also regarded as valid for Switzerland.

#### 2.2.1. Health-Literacy-Index and Specific Indices

A Health-Literacy-Index was built that included all 47 items. Respondents were asked to rate the perceived difficulty of different aspects concerning accessing, understanding, appraising, and applying health information. The degree of difficulty was assessed with a 4-point Likert scale and numerical values were accordingly assigned as follows: “very easy” = 4; “rather easy” = 3; “rather difficult” = 2; “very difficult” = 1. The index was only calculated if a minimum respondent rate of 80% in all 47 items was achieved. To allow for better comparisons, the index was assigned a common metric between 0 and 50. The following formula, defined in the HLS-EU study [6] and in the HLS_15_-CH study, was applied:

$$Index=\left(mean-1\right)\times \frac{50}{3}$$

The Health-Literacy-Index was consequently interpreted as recommended by the HLS-EU-consortium [5]. Three threshold values were defined and resulted in the following four categories:

- 0 to 25 points = inadequate health literacy,
- >25 to 33 points = problematic health literacy,
- >33 to 42 points = sufficient health literacy,
- >42 to 50 points = excellent health literacy.

Internal consistency of the instrument was high (Cronbach’s alpha = 0.889).

The 47 items of the HLS-EU-Q47 survey allowed for the calculation of three different sub-indices of 15 or 16 items each for the domains of healthcare, disease prevention and health promotion. In particular, the items Q1.1–Q1.16 were used to calculate the healthcare index, Q1.17–Q1.31 for the disease prevention index, and Q1.32–Q1.47 for the health promotion index. This is in line with other studies (e.g., [6,7]), in order to allow for the comparability of the results. Please see Table A1 for an overview of all the items.

#### 2.2.2. Sociodemographic Characteristics

The sociodemographic variables included were age, gender, education, financial deprivation, self-perceived social status, migration background and type of settlement. Education was re-categorized according to the International Standard Classification of Education (ISCED) and consequently divided into the three categories, i.e., low (level 0–2), medium (level 3–4) and high (level 5–6) education. Migration background was defined as having at least one parent who was born abroad. Financial deprivation was measured according to the HLS-EU with three items and included the ability to afford to see a doctor, the ability to pay for medication and general problems when paying bills [6]. A positive factor score indicated higher financial deprivation, and a negative score consequently lower financial deprivation. Finally, the self-perceived social status varied on a scale from 1 = lowest status in society to 10 = highest status in society.

### 2.3. Statistical Analyses

Collected data were weighted according to the sociodemographic characteristics age/gender interlocked, type of settlement and highest level of education to account for the sample design, to adjust for respective sociodemographic characteristics and to enhance the representativity of the results. The Federal Statistical Office’s statistics served as a reference for the weights [11,12]. A descriptive statistical analysis was used to characterize the sample and to analyze the frequency of answers regarding health-literacy associated items. Bivariate analyses were performed to investigate associations between health literacy and sociodemographic characteristics, as well as multiple regression models to measure the effects of selected sociodemographic characteristics on health literacy. To investigate the associations between the Health-Literacy-Index and sociodemographic variables, Chi-squared test were used. In a second step, after testing the Index for normal distribution, any associations between the Health-Literacy-Index and sociodemographic characteristics (independent variables) were assessed with Spearman’s rank correlation coefficients and a standardized regression model. Here, Health-Literacy Index, age, education (ISCED levels), self-perceived financial deprivation, and self-assessed social status were included as metric variables, whereas gender and migration were labeled as dummy variables. The same model applied in the HLS-EU study, which explains health literacy based on gender, age, education, financial deprivation, and social status, was calculated [6].

A statistical analysis was conducted using the IBM SPSS v. 26 software (IBM Corp., Armonk, NY, USA). The results were considered statistically significant based on the threshold value *p* ≤ 0.05.

## 3. Results

### 3.1. Characteristics of the Study Population

Overall, 1000 participants were included in the analysis. The mean age of study participants was 46.1 (SD 17.9) years. The youngest respondent was 18 and the oldest participant was 88 years old. Further sociodemographic characteristics are presented in Table 1.

### 3.2. Health Literacy of the Population of Zurich

**Figure 1:**
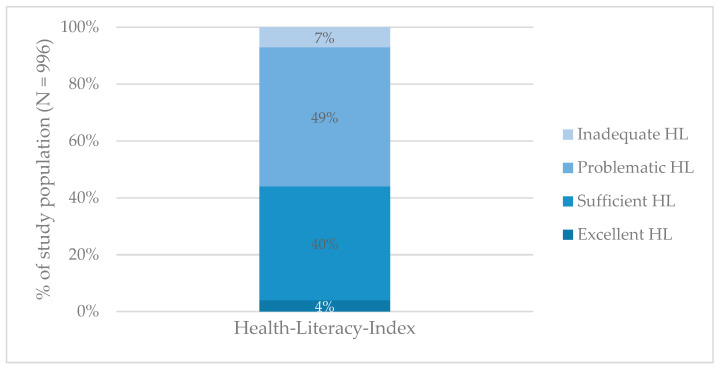
Health literacy (HL) levels among the study population.

The Health-Literacy-Index was calculated for *n* = 996. The survey of the residents of the canton of Zurich shows that more than half of the respondents have low health literacy levels (56%), representing either problematic or inadequate health literacy (Figure 1).

Most difficulties were reported in the area of health promotion, where 62% of the respondents reported low health literacy (49% problematic and 13% inadequate). With regard to the four steps of information processing–finding, understanding, judging, and applying–the results show that the respondents generally had more difficulties in appraising information (34% problematic and 34% inadequate). Regarding single items, the main difficulties were reported in finding information on political changes that may affect health, whereby 61% found it rather or very difficult (Table A1). Furthermore, difficulties were also reported in judging how one’s own living environment affects health and wellbeing, as 39% of the respondents considered this to be difficult. Difficulties were also reported in the area of disease prevention, where 44% reported problematic and 11% reported inadequate health literacy. Here, respondents reported great difficulties in judging whether the information on health risks in the media is reliable, and 45% found it rather difficult or very difficult. Furthermore, 37% of the respondents reported problematic (30%) or inadequate (7%) health literacy in in the domain of health care. Here, for 50% of the respondents, it was rather or very difficult to judge the advantages and disadvantages of different treatment options. Further findings about the different domains of health literacy are reported in Table A2.

### 3.3. Sociodemographic Characteristics and Health Literacy

Considering sociodemographic characteristics, the results show that in the canton of Zurich 42% of women and 46% of men were reported to have sufficient or excellent health literacy (Chi-squared (3) = 3.4, *p* = 0.340, *n* = 996). However, the association is not found to be significant (*CC* = 0.058, *p* = 0.340; Cramer’s *V* = 0.058, *p* = 0.340) (Table 2). With regards to the different age groups, the findings show that health literacy also varies according to different age groups (Chi-squared (6) = 105.7, *p* < 0.000, *n* = 996). However, the association is not very strong (*CC* = 0.310, *p* < 0.000; Cramer’s *V* = 0.230, *p* < 0.000), as 17% of people with a low level of education reported inadequate, and 55% problematic health literacy, compared to 3% and 40% of those with a high educational level (Chi-squared (6) = 51.5, *p* < 0.000, *n* = 996). The association is not very strong (*CC* = 0.222, *p* < 0.000; Cramer’s *V* = 0.161, *p* < 0.000). Migration background has no influence on health literacy levels (Chi-squared (3) = 2.0, *p* = 0.571, *n* = 996; *CC* = 0.045, *p* = 0.571; Cramer’s *V* = 0.045, *p* = 0.571). In addition, three quarters (75%) of the respondents living in rural areas reported problematic or inadequate health literacy, whereas among people living in the city 54% reported problematic or inadequate health literacy (Chi-squared (6) = 12.7, *p* = 0.048, *n* = 996), with a rather weak association (*CC* = 0.112, *p* = 0.048; Cramer’s *V* = 0.0.08, *p* = 0.048). Considering the small number of respondents living in rural areas, these percentages might not be representative for the rural population. Concerning self-perceived social status, there is an association in relation to health literacy (Chi-squared (6) = 53.3, *p* < 0.000, *n* = 989). The association is not very strong (*CC* = 0.226, *p* < 0.000; Cramer’s *V* = 0.164, *p* < 0.000). Moreover, almost two thirds (72%) of the people with low financial deprivation reported adequate health literacy levels, whereas among people with a very high financial deprivation, the majority (76%) reported low health literacy (Chi-squared (12) = 182.688, *p* < 0.000, *n* = 962). This association is again not very strong (*CC* = 0.399, *p* < 0.000; Cramer’s *V* = 0.252, *p* < 0.000).

The highest correlation of sociodemographic characteristics and health literacy emerged with financial deprivation (r = −0.408) (Table 3). In addition, a positive correlation between self-perceived social status and health literacy (r = 0.288) was identified as well as with education (r = 0.260). Considering different age groups, the results show that health literacy decreases with increasing age (r = −0.209). Although weak, all these correlations are significant. Migration background has only a weak negative effect (r = −0.077), and there is no statistically significant correlation with gender.

The Health-Literacy-Index (dependent variable) was tested for normal distribution. The findings indicate a peaked normal distribution, with a skewness 0.098 and kurtosis of 0.658. The multiple regression model explains almost 20% (R^2^ = 0.203) of the total variance in health literacy (Table 4). Even though it is rather weak, financial deprivation shows the greatest impact among the included sociodemographic variables: the greater the financial deprivation, the greater the difficulties in dealing with health information, and the lower health literacy. According to these findings, financial deprivation (β = −0.290, 95% CI: −1.793–−1.121) is the strongest predictor of health literacy. The second strongest factor is education (β = 0.128, 95% CI: 0.226–0.794), as Education level has a positive influence on health literacy. The correlation between health literacy and self-perceived social status is weak (β = 0.078, 95% CI: 0.013–0.525). On average, health literacy increases with higher social status. In contrast, age has a weak, negative influence (β = −0.119, 95% CI: −0.050–−0.017. Finally, migration (β = −0.26, 95% CI: −0.850–0.319) and gender (β = 0.056, 95% CI: −0.008–1.142) were found to be the weakest of the six determinants tested, and do not have a significant influence on health literacy.

## 4. Discussion

The first study on health literacy among the residents of the canton of Zurich shows that more than half (56%) reported low, i.e., problematic or inadequate health literacy. Thus, a significant proportion of the population seems to have difficulties in managing health information, particularly with regard to appraising health information.

When comparing the results with the Swiss data of 2015 (HLS_15_-CH), it appears that, on average, the population of Zurich tends to have nearly the same health literacy levels as the Swiss population. Indeed, 45% of the Swiss population reported problematic health literacy and 9% reported inadequate health literacy, compared to 49% and 7% reported by the respondents of the canton of Zurich. The interviewed residents of Zurich expressed greatest difficulties in the area of health promotion, where they reported slightly more pronounced difficulties in managing health information compared to the Swiss population (mean ZH 31.6 vs. CH 32.7) [7]. Finding information about political changes that may affect health, seems to present a particularly difficult challenge. Almost two thirds of the respondents reported difficulties (61 vs. 44% CH found it rather or very difficult) [7]. These difficulties could be related to confusion and uncertainties regarding cantonal health politics. Greater difficulties are also evident in Zurich when it comes to judging how one’s own living environment affects one’s own health and wellbeing (39% vs. CH 28%) [7]. These differences could be amplified by the fact that Zurich is, comparatively, a rather urban canton with special living conditions that potentially differ from the ones of other cantons, potentially due to its high population density and because of the relatively high availability of health and medical services.

Recently, a different study on health literacy of the Swiss population (HLS_19–21_-CH) was conducted [3]. It showed that health literacy among the Swiss population has slightly decreased from 2015 to 2020. Compared to this recent study, the residents of the canton of Zurich reported greater difficulties in dealing with health information than the Swiss population, two years later. However, the comparability of the findings of this study in Zurich and the HLS_15_-CH with the recent HLS_19–21_-CH study is limited, as several aspects such as the questionnaire itself, the interview methods and the calculation methods of the Health-Literacy-Index were modified [3]. Nevertheless, comparisons are possible at the level of certain individual items. On an item level, there are some cantonal and national similarities as well as differences. Moreover, Swiss studies in 2015 and 2020 both showed differences in health literacy between the three main language regions [3,7]. Residents from the French-speaking part reported slightly higher health literacy levels than those in the German-speaking part, which in turn reported less difficulties than people from the Italian-speaking part of Switzerland. Hence, the small differences in health literacy between the cantonal and the national data might be explained by the differences in language regions. Taken together, these results emphasize the importance of focusing research and improvement measures not only on a national but also on a regional or cantonal level, especially in a country such as Switzerland, where the healthcare system is strongly diversified and is based on a federalist structure.

The results from this study are not only comparable to Switzerland, but also to findings in other European countries. In Germany for example, 58.8% of the population reported low health literacy, and most difficulties have been found in the area of health promotion [5,13]. A former comparative study in Europe [4] reported that more than 10% of the total population had inadequate levels of health literacy, with a proportion ranging between 1.8% (the Netherlands) and 26.9% (Bulgaria). Thus, the insufficient health literacy of the population presents a challenge that needs to be addressed on several levels by public health efforts on a continental, national as well as on a regional level, such as in the canton of Zurich.

The present results show that health literacy is not just a problem of a minority, approximately every second person seems to have difficulties with finding, understanding, appraising, and applying health information. Nevertheless, the findings confirm that health literacy follows a social gradient [14,15,16] and there are some specific population groups that represent a higher proportion of low health literacy. The highest predictor is financial deprivation, as the higher financial deprivation is, the lower the health literacy. This is true for Zurich as well as Switzerland. The same could be found along education level and self-reported social status; the lower the educational level and social status, the lower health literacy. These findings confirm what has been found on the national level [3,7], as well as in other European countries [4,5,6,13,17,18,19,20,21].

The results of the present study stress the necessity for further research and evidence-based interventions to strengthen health literacy. A particular need for action becomes apparent for certain population groups, since these groups are already exposed to greater health burdens and are therefore often described as vulnerable. In general, these population groups often have poorer health status, a less healthy lifestyle, and at the same time, frequently report low health literacy [3,5,19,20,22,23]. For this reason, the need to strengthen the health literacy of these population groups can be described as urgent or a priority.

One avenue to support these population groups in managing health information could be to develop interventions on the local or cantonal level. Such an approach may offer a comparatively short-term and low-threshold opportunity to develop measures close to or together with the affected population. Moreover, it would help to specifically manage problems that are common in a certain locality. In addition, local approaches can be evaluated with relatively little effort, and, when successful, can be systematically scaled and implemented on a greater level. Nonetheless, national and international measures and actions remain fundamental to strengthen the health literacy of populations.

There are some limitations that need to be considered when interpreting the present findings. First, all data collected were self-reported and based on a subjective assessment of partially hypothetical events and not on an objective observation. Therefore, there might be a risk of a reporting bias and of social desirability bias, and the level of health literacy could have been under- or overestimated. However, respondents were not asked to judge their health literacy, but to report their self-perceived difficulties in dealing with health information, and there is no evident reason for them to have misreported difficulties with respect to health information. Irrespective of this, it can be stated that these self-reported difficulties require special attention. Additionally, quota and inclusion criteria may have only partially allowed for an unbiased selection of participants, as interviewers were free to choose the location of recruitment. Furthermore, the survey was conducted in German, which is the official and most widely used language in the canton of Zurich. However, this might have excluded some people less competent in this language, for example people with a migration background. For this reason, we suggest further research in less easily accessible population groups and people less competent in the German language. Therewith the challenges faced by these individuals could be tackled in a systematic way and better addressed with tailored interventions. Finally, as already indicated above, several methodological differences between the different studies on health literacy limit the comparability of these results with each other. Nonetheless, the broad approach to health literacy using a validated instrument should be noted as a strength of the present study and several others, as health literacy was examined along the three dimensions of healthcare, health promotion and disease prevention, which allowed a variety of aspects to be considered in order to understand which aspects present more difficulties for the population in managing health information.

## 5. Conclusions

The present findings show that a large part of the population of the canton of Zurich reported difficulties in dealing with health information. Certain population groups had even greater difficulties, and correspondingly reported lower health literacy levels. Therefore, the need for action to strengthen health literacy of these population groups can be described as urgent or a priority. Measures at a local or regional level are needed as urgently as overarching and coordinated efforts at the national and international level. The present study on the cantonal level indicates that future studies on health literacy should consider cultural factors such as linguistic regions or the political system. Based on our findings, more specific and tailored interventions could be created—first on a local and relatively low level, and then implemented on greater levels. Furthermore, measures to promote health literacy should be implemented at the individual level as well as at the level of organizations and systems. Health literacy is not only the responsibility of the individuals, but is also strongly determined by external conditions and the societal context in which individuals live [24,25,26], as the present findings also confirmed. Finally, it is important to verify whether the measures taken have led to the intended effect by regular monitoring on the respective level (regional, national, international).

## Figures and Tables

**Table 1:** Sociodemographic characteristics of the sample.

	Total (*n* = 1000) % (*n*)
Gender	
Female	51% (507)
Male	49% (493)
Age	
18–39	37% (374)
40–64	42% (420)
65+	21% (206)
Education	
Low	7% (72)
Medium	74% (739)
High	19% (189)
Migration background	
Yes	40% (398)
No	60% (602)
Type of settlement	
Rural	5% (50)
Small/mid-sized city	16% (160)
Big city	79% (790)
Self-perceived social status	
Until lower intermediate	37% (374)
Intermediate	35% (349)
From upper intermediate	27% (270)
Financial deprivation	
Very low	16% (163)
Low	17% (166)
Middle	19% (192)
High	8% (76)
Very high	38% (369)

(*n*) unweighted number of cases. The total can differ from *n* = 1000 due to missing values. Percentages are rounded mathematically and do not always add up to exactly 100%.

**Table 2:** Health literacy by sociodemographic factors.

	Excellent	Sufficient	Problematic	Inadequate
	% (*n*)	% (*n*)	% (*n*)	% (*n*)
**Gender (*n* = 996)**				
Male (*n* = 490)	2% (11)	44% (210)	45% (241)	8% (28)
Female (*n* = 506)	6% (17)	36% (196)	53% (269)	5% (24)
**Age (*n* = 996)**				
18–39 (*n* = 373)	2% (6)	47% (173)	47% (183)	5% (11)
40–64 (*n* = 419)	6% (17)	43% (188)	49% (209)	1% (5)
65+ (*n* = 204)	3% (5)	21% (45)	55% (118)	22% (36)
**Education (*n* = 996)**				
Low (*n* = 71)	2% (1)	26% (19)	55% (39)	17% (12)
Medium (*n* = 736)	2% (13)	40% (293)	54% (396)	5% (34)
High (*n* = 189)	8% (14)	49% (94)	40% (75)	3% (6)
**Migration background (*n* = 996)**				
Yes (*n* = 397)	4% (11)	36% (155)	51% (206)	9% (25)
No (*n* = 599)	4% (17)	43% (251)	48% (304)	5% (27)
**Type of settlement (*n* = 996)**				
Rural (*n* = 50)	0% (0)	26% (13)	70% (36)	4% (1)
Small/mid-sized city (*n* = 160)	6% (7)	35% (59)	51% (84)	8% (10)
Big city (*n* = 786)	4% (21)	42% (334)	48% (390)	7% (41)
**Self-perceived social status (*n* = 989)**				
Until lower intermediate (*n* = 371)	2% (7)	29% (115)	55% (215)	13% (34)
Intermediate (*n* = 348)	1% (5)	47% (155)	48% (175)	4% (13)
From upper intermediate (*n* = 270)	7% (16)	46% (134)	44% (115)	3% (5)
**Financial deprivation (*n* = 962)**				
Very low (*n* = 161)	12% (18)	51% (87)	36% (54)	2% (2)
Low (*n* = 166)	6% (5)	65% (115)	26% (41)	3% (5)
Middle (*n* = 192)	2% (3)	40% (77)	55% (107)	3% (5)
High (*n* = 76)	1% (2)	30% (26)	59% (44)	10% (4)
Very high (*n* = 367)	0% (0)	24% (92)	63% (240)	13% (35)

(*n*) unweighted number of cases. The total number of results may differ from *n* = 1000 due to missing values. Percentages are weighted and rounded mathematically and do not always add up to exactly 100%.

**Table 3:** Correlations between Health-Literacy-Index and sociodemographic characteristics.

Health-Literacy-Index and…	*ρ*
Gender	−0.023
Age	−0.209 *
Education	0.260 *
Financial deprivation	−0.408 *
Self-perceived social status	0.288 *
Migration background	−0.077 *

* The correlation is significant at 0.01 level (two-sided).

**Table 4:** Regression model of the Health-Literacy-Index and sociodemographic characteristics.

Health-Literacy-Index and…	β
Gender	0.056
Age	−0.119 **
Education	0.128 **
Financial deprivation	−0.290 **
Self-perceived social status	0.078 *
Migration background	−0.26
*Adjusted R* ^2^	*0.203*

* The correlation is significant at 0.05 level; ** significant at 0.01 level.

## Data Availability

Participants of this study did not consent to their data being publicly shared, so supporting data is not available.

## Appendix A

**Table A1:** HLS-EU-Q47 items by responses of the sample (in%).

Item	On a Scale from Very Easy to Very Difficult, how Easy Would You Say It Is…	Very Easy	Rather Easy	Rather Difficult	Very Difficult	No Answer
1	to find information about symptoms of illnesses that concern you?	2	15	58	25	0
2	to find information on treatments of illnesses that concern you?	4	19	56	20	1
3	to find out what to do in case of a medical emergency?	2	13	47	37	1
4	to find out where to get professional help when you are ill? (e.g., doctor, pharmacist, psychologist)	1	13	55	31	0
5	to understand what your doctor says to you?	4	12	54	30	0
6	to understand the leaflets that come with your medicine?	6	16	50	26	2
7	to understand what to do in a medical emergency?	1	10	54	33	2
8	to understand your doctor’s or pharmacist’s instruction on how to take a prescribed medicine?	0	5	54	41	0
9	to judge how information from your doctor applies to you?	2	19	56	22	1
10	to judge the advantages and disadvantages of different treatment options?	15	35	37	11	2
11	to judge when you may need to get a second opinion from another doctor?	9	29	43	14	5
12	to judge if the information about illness in the media is reliable? (e.g., TV, Internet or other media)	17	27	43	9	4
13	to use information the doctor gives you to make decisions about your illness?	4	13	60	21	2
14	to follow the instructions on medication?	2	5	51	42	0
15	to call an ambulance in an emergency?	0	2	32	65	1
16	to follow instructions from your doctor or pharmacist?	1	4	54	41	0
17	to find information about how to manage unhealthy behavior such as smoking, low physical activity and drinking too much?	1	11	53	32	3
18	to find information on how to manage mental health problems like stress or depression?	6	29	41	18	6
19	to find information about vaccinations and health screenings that you should have? (e.g., colorectal cancer screening, blood sugar test)	3	18	54	23	2
20	to find information on how to prevent or manage conditions like being overweight, high blood pressure or high cholesterol?	1	12	60	27	0
21	to understand health warnings about behavior such as smoking, low physical activity and drinking too much?	1	11	52	36	0
22	to understand why you need vaccinations?	3	11	54	30	2
23	to understand why you need health screenings? (e.g., colorectal cancer screening, blood sugar test)	1	6	50	43	0
24	to judge how reliable health warnings are, such as smoking, low physical activity and drinking too much?	2	16	48	33	1
25	to judge when you need to go to a doctor for a check-up?	6	22	49	23	0
26	to judge which vaccinations you may need?	13	29	43	14	1
27	to judge which health screenings you may have? (e.g., colorectal cancer screening, blood sugar test)	17	29	34	19	1
28	to judge if the information on health risks in the media is reliable? (e.g., TV, Internet or other media)	20	25	44	9	2
29	to decide if you should have a flu vaccination?	5	19	46	28	2
30	to decide how you can protect yourself from illness based on advice from family and friends?	6	16	59	16	3
31	to decide how you can protect yourself from illness based on information in the media? (e.g., Newspapers, TV or Internet)	10	28	47	10	5
32	to find information on healthy activities such as exercise, healthy food and nutrition?	1	3	57	39	0
33	to find out about activities that are good for your mental well-being?	4	18	49	26	3
34	to find information on how your neighborhood could be more health-friendly?	6	30	45	18	1
35	to find out about political changes that may affect health?	22	39	29	7	3
36	to find out about efforts to promote your health at work, at school or in the community?	6	21	42	20	11
37	to understand advice on health from family members or friends?	4	11	60	23	2
38	to understand information on food packaging?	9	21	51	18	1
39	to understand information in the media on how to get healthier?	8	17	54	19	2
40	to understand information on how to keep your mind healthy?	4	23	53	19	1
41	to judge where your life affects your health and wellbeing?	4	35	45	15	1
42	to judge how your housing conditions help you to stay healthy?	3	22	56	18	1
43	to judge which everyday behavior is related to your health?	2	25	48	25	0
44	to make decisions to improve your health?	4	18	52	26	0
45	to join a sports club or exercise class if you want to?	2	10	47	40	1
46	to influence your living conditions that affect your health and wellbeing?	5	29	49	17	0
47	to take part in activities that improve health and wellbeing?	4	21	52	19	4

Note: This study used a translated version of this questionnaire originally presented in the German language. Please note that, to allow for comparability, the same questionnaire has been used in various studies (e.g., [5,6,7]), and copyright is not violated.

## Appendix B

**Table A2:** Health literacy (HL) levels among the study population by domain.

Domains of HL	Excellent	Sufficient	Problematic	Inadequate
health literacy	4%	40%	49%	7%
health promotion	3%	35%	49%	13%
disease prevention	6%	39%	44%	11%
healthcare	7%	56%	30%	7%
